# Understanding the Drivers of CAB PrEP Uptake and Use among Women in sub-Saharan Africa to Build Demand for New PrEP Methods

**DOI:** 10.1007/s11904-024-00715-y

**Published:** 2024-12-07

**Authors:** Casey Bishopp, Zoe Mungai-Barris, Elmari Briedenhann, Emily Donaldson, Elizabeth Irungu, Katie Schwartz

**Affiliations:** 1 Family Health International 360, Durham, NC USA; 2 Mann Global Health, Columbus, NC USA; 3 University of the Witwatersrand, Johannesburg, South Africa; 4 Jhpiego, Nairobi, Kenya

**Keywords:** Cabotegravir, PrEP, Sub-Saharan Africa, Women, HIV prevention, Injectable

## Abstract

**Purpose of Review:**

As injectable cabotegravir for pre-exposure prophylaxis (CAB PrEP) is introduced in sub-Saharan Africa, it is important to understand how behavioral drivers may influence women’s decisions around whether or not to use it.

**Recent Findings:**

Facilitating factors include prior familiarity with injections and the perceived efficacy of CAB PrEP, while barriers include a fear or dislike of needles and negative attitudes held by community members and influencers. Further research is needed to fully understand behavioral factors affecting African women’s CAB PrEP use.

**Summary:**

HIV prevention policymakers, practitioners, advocates, and clients are optimistic about CAB PrEP, predicting that this long-acting method will be popular among women in sub-Saharan Africa. However, women may also face barriers to use. Knowledge of behavioral facilitators and barriers can enhance the adaptation or development of HIV prevention communication and demand generation strategies that support informed decision-making in a multi-method market.

## Introduction

Global progress is being made against new HIV infections, and nowhere is this more pronounced than in sub-Saharan Africa. In 2022, the numbers of new infections specifically among young women was half that of 2010. Yet women and girls continue to be disproportionately affected across the region. In fact, new HIV infections are more than three times higher among adolescent girls and young women than among males of the same ages [1].

In 2012, the U.S. Food and Drug Administration (FDA) approved the first PrEP method (oral tenofovir-based PrEP). Despite high efficacy observed in randomized placebo-controlled trials among individuals who were able to use oral PrEP consistently and the clear benefits of primary prevention at the community level, the scale-up of oral PrEP has been slow [2, 3]. In 2022, an estimated 2.5 million people worldwide were using oral PrEP, far below the United Nations 2025 target of 10 million people [1].

Challenges with scale-up of oral PrEP include limited access, low community awareness, insufficient demand generation, inadequate provider training, and inadequate service delivery models to reach marginalized populations [4]. Given the daily dosing requirements during periods when potential exposure to HIV could occur, adherence to the regimen can be challenging. A global systematic review found that 41% of PrEP clients discontinued PrEP by six months, and 47% of them reinitiated PrEP within a year [5].

New PrEP methods are gradually gaining national regulatory approval across sub-Saharan Africa (SSA). The dapivirine vaginal ring (PrEP ring) was recommended by the World Health Organization (WHO) in January 2021 and has since been registered for use as HIV prevention in 11 African countries [6]. Most recently, injectable cabotegravir for HIV prevention (CAB PrEP) was approved by the FDA in December 2021 and recommended by WHO in July 2022 [7, 8]. Since FDA approval, the product has been registered in 17 countries, nine of which are in SSA [9].

The PrEP market is slowly moving from a single product to multi-method, similar to the family planning market. As the PrEP ring and CAB PrEP become available through implementation research studies or programmatic pilots, community awareness of these new methods and demand generation for PrEP are needed. Effective demand generation campaigns, communication strategies, and behavior change interventions need to be rooted in evidence-based principles of behavior change to be successful [10].

How people will use PrEP in a multi-method market remains unknown; to maximize new product introduction efforts, it is important to leverage what is known about facilitators and barriers to PrEP uptake. Much is understood and has been summarized for oral PrEP and, to a lesser extent, the PrEP ring [11, 12]. This paper summarizes what is known about barriers and facilitators to CAB PrEP uptake and use to inform the development of successful evidence-based demand generation and behavior change interventions for multi-method PrEP markets.

## Methodology

### Conceptual Framework

**Fig. 1:**
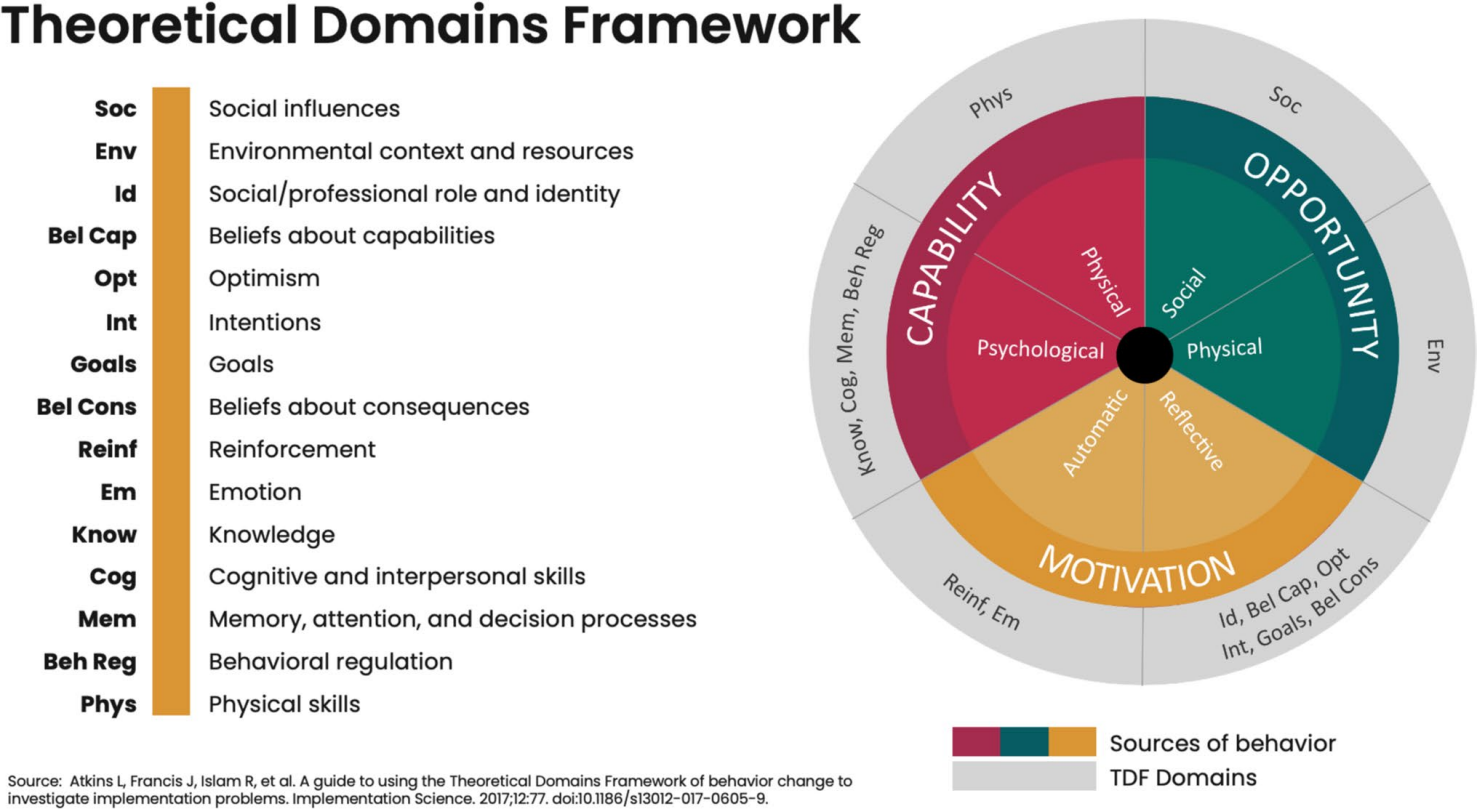
TDF as an extension of the COM-B model [14]

Applying a theoretical lens can help build the understanding of behavioral drivers needed to inform programming and promote behavior change [13, 14]. This review utilizes the Theoretical Domains Framework (TDF) and the Capability, Opportunity, Motivation, and Behavior model (COM-B) to provide a system for comprehending and addressing drivers of current and desired behavior in relation to CAB PrEP uptake. The COM-B model argues that an understanding of *capability* (the individual’s ability to perform a behavior), *opportunity* (environmental factors that influence the individual’s behavior), and *motivation* (the willingness of the individual to adopt a new behavior or change current behaviors) can be used to develop interventions that promote positive behavior change [15]. The TDF, illustrated in Fig. 1, is derived from 33 behavioral theories and 128 psychological constructs relevant to program implementation and further distilled into 14 behavioral domains. It builds on the elements of the COM-B model to reveal underlying barriers and facilitators of behavior change, offering an evidence-informed framework to understand and promote behavior change [15].

Informed by the TDF and COM-B model, this review identifies hypothetical and confirmed facilitators and barriers to CAB PrEP uptake among women in SSA with the aim of informing future research, programs, and implementation efforts in demand generation for CAB PrEP uptake. For the purposes of this review, the authors defined hypothetical and confirmed facilitators and barriers as follows:

1. **Hypothetical** behavioral factors: Barriers and facilitators derived from formative research (descriptive studies, human-centered design projects, and discrete choice experiments) conducted without product use, including perspectives of those who do not have CAB PrEP use experience, and applicable learnings from the rollout of other HIV prevention methods.
2. **Confirmed** behavioral factors: Barriers and facilitators documented in CAB PrEP clinical trials and other research involving CAB PrEP use by study participants.

### Review Design

This desk review of peer-reviewed journal articles and programmatic documents involving CAB PrEP use by women in SSA considered the following inclusion criteria to select literature for screening and review:

- Published in English from 2015 to 2023.
- Addresses outcomes related to behavioral drivers of CAB PrEP use, the general CAB PrEP implementation and demand generation landscape, or lessons derived from the rollout of other HIV prevention technologies potentially applicable to CAB PrEP, and.
- Reports findings from clinical trials, open label extension studies, implementation studies, descriptive studies, human-centered design research, discrete choice experiments, and other research methodologies.

The following key search terms were used to identify relevant literature: (“women” OR “adolescent girls” OR “young women”) AND (“drivers” OR “factors” OR “facilitators” OR “barriers”) AND (“Cabotegravir” OR “long-acting PrEP” OR “CAB PrEP” OR “CAB LA”) AND (“sub-Saharan Africa”) AND (“2015” [Date - Publication]: “2023” [Date – Publication]).

The search was completed on PubMed, Africa-Wide information, Global Health, Women’s Studies International, Scopus, Embase, Global Index Medicus (WHO), the Development Experience Clearinghouse (USAID), and several project websites, including HIV conference, HIV prevention, and social and behavior change project websites.

Search results were then assessed by screening titles of literature for relevance, followed by a review of abstracts and a full document review based on the inclusion criteria. Of the 120 peer-reviewed articles that were screened, 62 met the criteria for inclusion and were reviewed. Studies that were specific to men or did not include data from sub-Saharan Africa were not included in this review. Additionally, due to the limited available literature on this topic from sub-Saharan Africa, it was not possible to disaggregate the data by key population or age range.

### Data Synthesis

Literature was coded into recurring and notable themes. After thematic categorization of behavioral factors from the literature, we deductively characterized behavioral factors in accordance with TDF domains. Behavioral factors were further categorized as facilitators or barriers to CAB PrEP uptake and use and as hypothetical or confirmed factors.

**Fig. 2:**
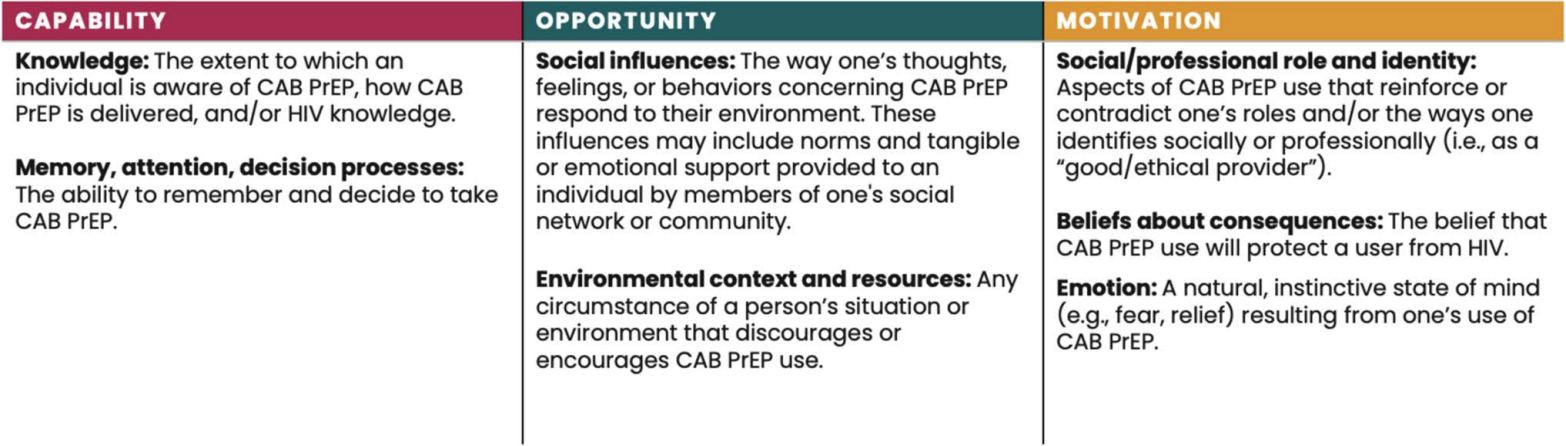
Definition of TDF domains as applied in this review

Seven TDF domains were identified and applied in this review: knowledge; memory, attention, and decision processes; social influences; environmental context and resources; social/professional role and identity; beliefs about consequences; and emotion. These domains are organized under the three COM-B pillars in Fig. 2.

## Key Insights

**Fig. 3:**
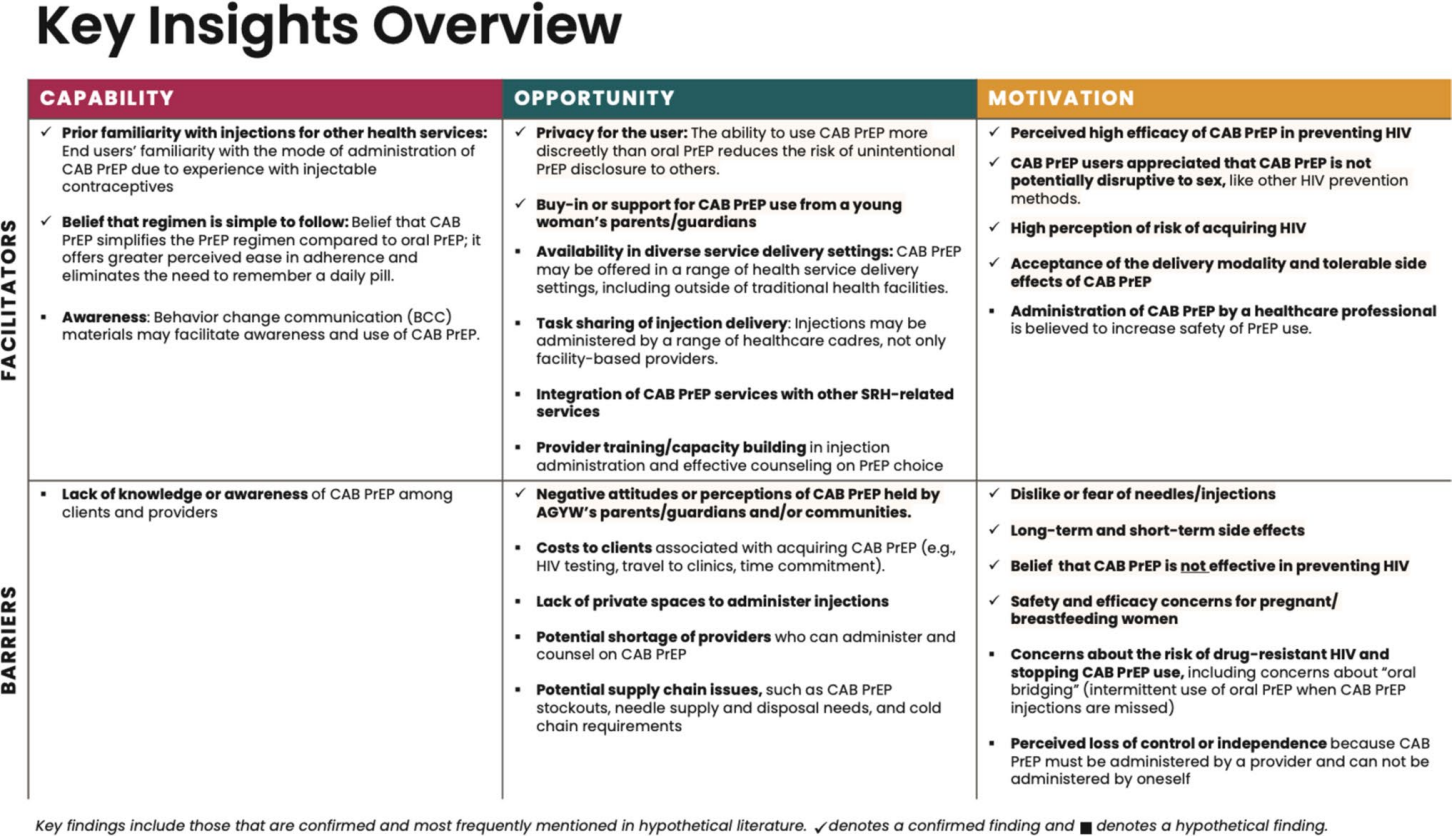
Key insights

Existing literature offers insights into confirmed and hypothetical factors likely to affect women’s use of CAB PrEP once it is widely available in African markets. These insights are organized along the COM-B model and TDF in Fig. 3.

### 1.) Capability [knowledge, memory, attention, decision processes].

#### Confirmed Factors

Confirmed capability factors include an individual’s prior familiarity with injectable contraceptives: an ability to use injectables for pregnancy prevention is considered an enabling factor for women to use CAB PrEP [16, 17]. Furthermore, end users believe that CAB PrEP simplifies the PrEP regimen (CAB is injected every two months compared to taking a daily pill). Unlike oral PrEP, which has to be carried with the user if they are mobile or traveling, CAB PrEP offers greater perceived ease in adherence, which is another confirmed factor [18–21].

#### Hypothetical Factors

A lack of knowledge and awareness of CAB PrEP as a long-acting HIV prevention method may act as a barrier to initial uptake and use. Information, education, and communication (IEC) materials may facilitate awareness and knowledge-building, which could ease this barrier and promote uptake of CAB PrEP [22]. High-quality materials may also help facilitate community sensitization and greater acceptance among key influencer groups of women end users, as well as correct myths and misinformation, which can be barriers [22–25]. Based on lessons from oral PrEP rollout, client- and provider-facing behavior change communication materials should emphasize the high efficacy and convenience of CAB PrEP, provide information on various PrEP options in non-technical language, and answer frequently asked questions [4]. Appointment reminders may also help facilitate continued use [4, 23].

### 2.) Opportunity [social influences, environmental context, and resources].

#### Confirmed Factors

CAB PrEP offers users discretion. Unlike oral PrEP, it does not need to be carried around, stored covertly in households, or used in settings where partners or family members may observe use.

 [16, 19, 20, 26–28]. CAB PrEP’s discreet nature helps prevent potential negative social consequences of unintentional PrEP disclosure. Concerns about and belief in myths about the harms of CAB PrEP, including that it causes infertility, changes in DNA, and/or cancer, are examples of negative perceptions in the minds of young women’s parents, guardians, and fellow community members that are barriers to use [29]. The presence of choice has been shown to increase uptake of all available PrEP methods, including CAB PrEP [30].

#### Hypothetical Factors

The effect of task sharing among healthcare personnel has yet to be formally explored, but studies posit that having injections administered by a range of healthcare cadres (including community, lay, and peer providers) and not exclusively by clinical physicians may result in less stigmatized services and reduce pressure on the health system [18, 23, 31, 32]. Similarly, offering CAB PrEP in a range of settings, including those outside traditional health facilities (e.g., pharmacies, food banks, schools, and mobile health units), could increase client reach, lower the burden of travel associated with traditional health settings, and destigmatize services [23, 31–35]. The integration of CAB PrEP services with other sexual and reproductive health (SRH)-related services could also act as a facilitator by reducing the number of clinic visits and travel costs for clients while easing the burden on the health system [4, 23, 25, 36, 37]. Finally, offering CAB PrEP at no charge to users and investing in healthcare provider training that focuses on correct administration of injections and nonjudgmental informed choice counseling could further facilitate uptake and continued use of this method [4, 18, 23, 24, 31, 38, 39]. On the other hand, barriers related to opportunity include potential supply chain issues, like CAB PrEP stockouts, cold chain requirements, or lack of sufficient needles [6, 18, 23, 33, 37, 40]. Where there is not a designated space in which to administer injections, women may find this lack of privacy to be a barrier [18, 23, 33, 36].

### 3.) Motivation [social/professional role and identity, beliefs about consequences, emotion].

#### Confirmed Factors

The perceived high efficacy of CAB PrEP to prevent HIV in most cases is extremely meaningful for women [16, 19–21, 27, 28, 34]. For some, this perception is due to beliefs that injections are more effective than other forms of medication; for others, it is because CAB PrEP becomes effective quickly after administration [17, 20, 40–43]. CAB PrEP users also appreciate that the method is not potentially disruptive to sex, unlike other HIV prevention methods.

One study conducted in several countries in SSA found that women who chose CAB PrEP over oral PrEP appeared to be at higher risk of HIV because they were less likely to live with a partner and more likely to have experienced recent physical intimate partner violence and/or to have been paid for sex [21, 27, 28, 41]. In these instances, a non-disruptive method was preferred. Overall, CAB PrEP was found to be tolerable, with no discontinuation of product use due to adverse events and a general acceptability of the needle size (1 1/2 inch) and site of administration (gluteal muscle) [16].

However, a dislike or fear of needles as well as concern about possible side effects, including injection site pain, injection site reaction, nausea, headaches, and dizziness, should be noted as factors that act as barriers to CAB PrEP use [17, 19–21, 26, 27, 37, 39]. For some women, the CAB PrEP injection is seen as irreversible due to its systemic nature, which is a barrier [19, 26, 27]. In addition, desire for pregnancy arose in research as one reason some may not opt for CAB PrEP. Some concern exists about its safety and efficacy profile for pregnant and/or breastfeeding women because it is not yet approved for use by this population in all countries [39].

####  Hypothetical Factors

The belief that CAB PrEP prevents HIV from all types of sexual exposures may facilitate use [22, 44]. Requiring CAB PrEP to be administered by a provider and not by the user may cause end users to perceive CAB PrEP as safe because administration by a professional would ideally reduce chances of mistakes, thereby facilitating use [35, 42]. However, required administration by a provider could also create a sense of loss of independence and/or control in relation to HIV prevention medication use, which may act as a barrier [23, 25, 35]. Evidence from clinical trials found acceptability among women with the site of CAB PrEP administration; but hypothetical findings suggest that some may experience discomfort with the injection site, resulting in the perception that CAB PrEP injections are invasive [18].

The risk of developing drug-resistant HIV after stopping CAB PrEP or perceived difficulty of CAB PrEP discontinuation may be barriers to use, but this has yet to be confirmed. (In some settings, guidelines dictate that oral PrEP be taken for a period of time after CAB PrEP use stops and may be used between missed doses of CAB PrEP; this is known as “oral bridging” and may be difficult for those who already have continuation challenges with oral PrEP) [31–33, 35, 42, 45, 46].

### 4.) Key Remaining Gaps

**Fig. 4:**
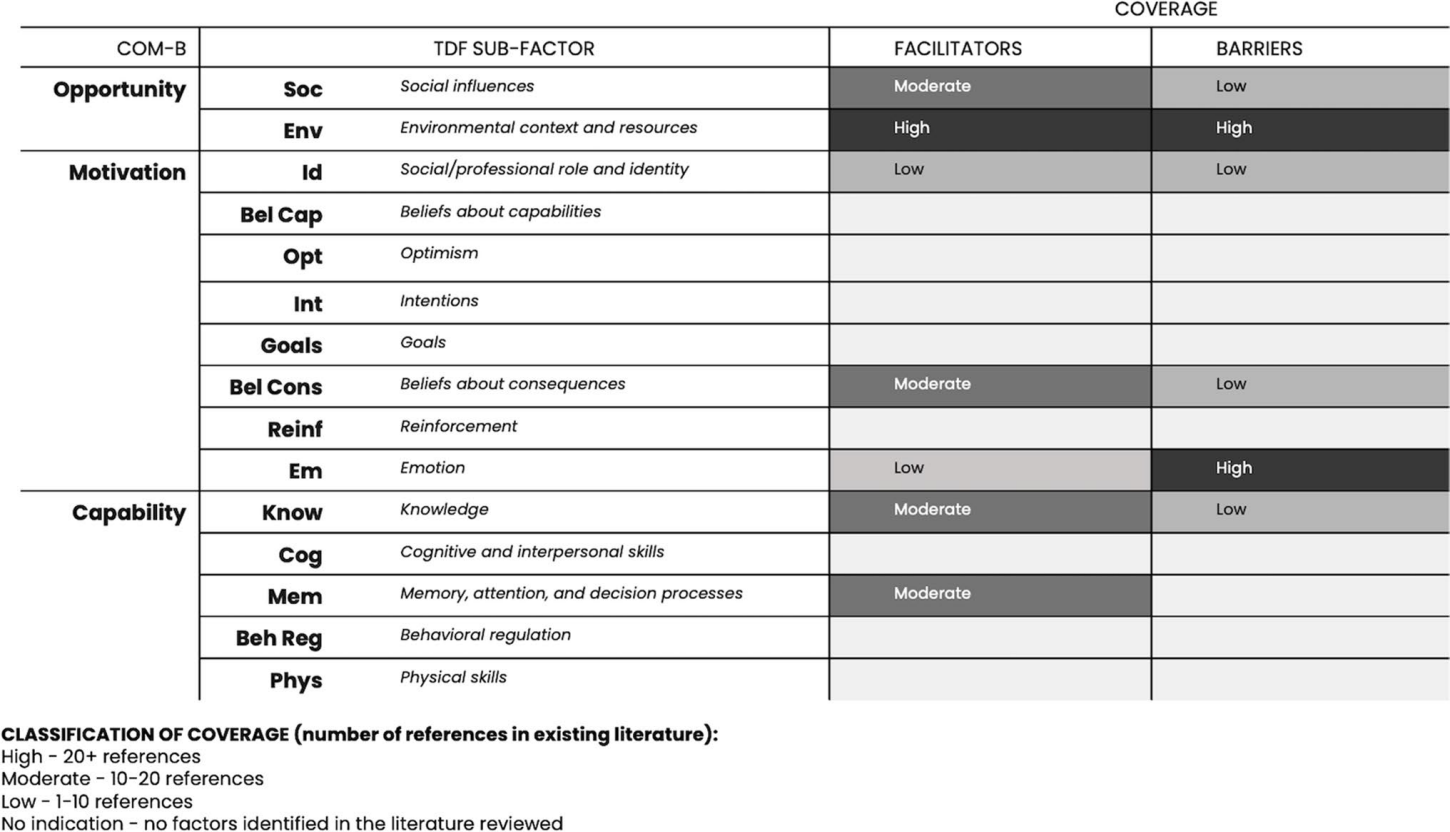
COM-B + TDF coverage and gaps

Further consideration and formative work are needed to address gaps in our understanding of women’s capability, opportunity, and motivation to use CAB PrEP. Our understanding of end-users’ motivation is particularly limited, and we do not yet fully understand some aspects of their capability. Figure 4 illustrates key gaps in the literature along the COM-B model and TDF.

Future efforts should endeavor to address research questions that speak to the capability, opportunity, and motivation of women in SSA to use CAB PrEP. Knowledge gaps must be identified and addressed; in particular, greater communication efforts are needed to mitigate the spread of misinformation about CAB PrEP on issues such as its effectiveness, the consequences of stopping CAB PrEP, and potential drug resistance. Equipped with the right information disseminated through channels with reach and influence, women in SSA may be more prepared to take up and use PrEP to stop the spread of HIV.

More work is needed to understand which factors affect women’s decision-making process when deciding whether or not to use PrEP generally and CAB PrEP specifically. Women are not a homogenous group, so the role of influencers must be explored to determine their impact on women’s decision to use CAB PrEP or not. For example, an understanding of the utility of peer-to-peer motivation is required to gauge what kind of user and influencer testimonials are most compelling. Exploratory work should be focused on the type of messages, specific communication strategies, and tactics implemented across a variety of channels that will be most successful in motivating women to seek out and adhere to CAB PrEP and HIV prevention services. Finally, researchers should explore whether CAB PrEP can be positioned in a manner that affirms control for the user—what are the unexplored emotional drivers behind women’s choice to use CAB PrEP?

To date, CAB PrEP has not yet been widely available in SSA and has been exclusively available in clinical and implementation study settings, with wider rollout expected in the future. Therefore, most of the available findings concerning drivers of CAB PrEP uptake and use are hypothetical, derived from formative research conducted through studies and projects that do not include product use outside of clinical and implementation study settings. Much of this work also took place prior to any national or large-scale rollouts of CAB PrEP in the general market. An opportunity exists to answer vital research questions as CAB PrEP becomes available to women in real-life rollout settings.

## Conclusions

A review of our findings along the TDF and COM-B model shows the evidence base is greatest related to opportunity factors; fewer data on capability—particularly on women’s knowledge of CAB PrEP—and a paucity of data on motivational factors are available. We found that emotions around CAB PrEP are considerably explored in existing literature, but other elements that might affect women’s motivation, such as optimism, intentions, goals, beliefs about capabilities, and reinforcement require further research.

Without knowing which factors facilitate or act as barriers to CAB PrEP use, national HIV prevention, combination prevention, and PrEP communication and demand generation strategies, campaigns, and plans will not be able to address women’s needs or communicate about CAB PrEP in a way that motivates them to act. Implementers can build more effective programs by aligning key behavioral facilitators and barriers with specific, evidence-based behavior change tactics to strengthen policies and programmatic interventions.

It is critical to understand the behavioral drivers of women’s use of PrEP to design demand generation efforts for them in the context of a multi-method market. Implementers must update strategies and communication efforts with product-specific behavioral insights to be truly comprehensive of all products—a need this review aims to address with regard to CAB PrEP. Our hope is that this examination of existing research on behavioral drivers can help shape future research agendas by consolidating what we know, illuminating what is still unknown, and emphasizing the importance of finding answers to questions about behavior as countries introduce CAB PrEP. 

## Key Reference

- Celum C, Grinsztejn B, Ngure K, et al. Preparing for long-acting PrEP delivery: building on lessons from oral PrEP. J Int AIDS Soc. 2023;26(S2): e26103.

- This study applied lessons about demand generation from oral PrEP to future CAB PrEP rollout.

- Grimsrud A, Wilkinson L, Delany-Moretlwe S, et al. The importance of the “how”: the case for differentiated service delivery of long-acting and extended delivery regimens for HIV prevention and treatment. J Int AIDS Soc. 2023;26(S2): e26095.

- This paper describes the importance of taking into account service delivery considerations and client needs during early implementation of long-acting injectable PrEP products.

- Liu AY, Buchbinder SP. CROI 2023: Epidemiologic trends and prevention for HIV and other sexually transmitted infections. Top Antivir Med. 2023;31(3):468–492.

- This study (HPTN-084-01) detailed behavioral factors associated with CAB PrEP decision-making from a clinical setting.

- Montgomery ET, Atujuna M, Krogstad E, et al. The invisible product: preferences for sustained-release, long-acting pre-exposure prophylaxis to HIV among South African youth. J Acquir Immune Defic Syndr. 2019;80(5):542–550.

- This paper outlines the HIV product preferences of 95 young South Africans and highlights the element of discretion as a key reason why young people may appreciate injectable PrEP.

- Moyo E, Murewanhema G, Musuka G, et al. Long-acting injectable drugs for HIV-1 pre-exposure prophylaxis: considerations for Africa. Trop Med Infect Dis. 2022;7(8):154.

- This study detailed considerations for CAB PrEP in wider rollout— specifically, provider training, community sensitization to CAB PrEP, and mitigating stress to health systems.

- Hosek S. CAB LA for HIV prevention in African cisgender female adolescents (HPTN 084-01). Paper presented at: Conference on Retroviruses and Opportunistic Infections; 2023; Seattle.

- This study’s primary objective was to evaluate the safety, tolerability, and acceptability of CAB PrEP in young women. Secondary objectives included examining adherence, safety, and efficacy.

## Data Availability

No datasets were generated or analysed during the current study.
